# Syntheses and crystal structures of bis­(4-methyl­pyridine-κ*N*)bis­(seleno­cyanato-κ*N*)zinc(II) and *catena*-poly[[bis­(4-methyl­pyridine-κ*N*)cadmium(II)]-di-μ-seleno­cyanato-κ^2^ *N*:*Se*;κ^2^ *Se*:*N*]

**DOI:** 10.1107/S2056989023000920

**Published:** 2023-02-07

**Authors:** Christian Näther, Inke Jess

**Affiliations:** a Institut für Anorganische Chemie, Universität Kiel, Max-Eyth Str. 2, 24118 Kiel, Germany; University of Aberdeen, United Kingdom

**Keywords:** synthesis, crystal structure, Zn(NCSe)_2_, Cd(NCSe)_2_, coordination compounds, 4-methyl­pyridine

## Abstract

The reactions of Zn(NO_3_)_2_^.^6H_2_O and Cd(NO_3_)_2_^.^4H_2_O with KSeCN and 4-meth­yl­pyridine (C_6_H_7_N; 4-picoline) lead to the formation of crystals of bis­(4-methyl­pyridine-κ*N*)bis­(seleno­cyanato-κ*N*)zinc(II), [Cd(NCSe)_2_(C_6_H_7_N)_2_] (**1**), and *catena*-poly[[bis­(4-methyl­pyridine-κ*N*)cadmium(II)]-di-μ-seleno­cyanato-κ^2^ *N*:*Se*;κ^2^ *Se*:*N*], [Cd(NCSe)_2_(C_6_H_7_N)_2_]_*n*_ (**2**), suitable for single-crystal X-ray diffraction. The asymmetric unit of compound **1** consists of one Zn cation that is located on a twofold rotation axis as well as one seleno­cyanate anion and one 4-methyl­pyridine ligand in general positions. The Zn cations are tetra­hedrally coordinated by two terminal N-bonding thio­cyanate anions and two 4-methyl­pyridine ligands, forming discrete complexes. The asymmetric unit of compound **2** consists of two crystallographically independent Cd cations, of which one is located on a twofold rotation axis and the second on a center of inversion, as well as two crystallographically independent seleno­cyanate anions and two crystallographically independent 4-methyl­pyridine ligands in general positions. The Cd cations are octa­hedrally coordinated by two N- and two S-bonding seleno­cyanate anions and two 4-methyl­pyridine ligands and are linked into chains by pairs of seleno­cyanate anions. Within the chains, the Cd cations show an alternating *cis*–*cis*–*trans* and *all*-*trans* coordination and therefore, the chains are corrugated. PXRD investigations prove that the Zn compound was obtained as a pure phase and that the Cd compound contains a very small amount of an additional and unknown phase. In the IR spectrum of **1**, the CN stretching vibration of the seleno­cyanate anion is observed at 2072 cm^−1^, whereas in the **2** it is shifted to 2094 cm^−1^, in agreement with the crystal structures.

## Abstract 2

In the crystal structures of the title compounds, the Zn cations are tetra­hedrally coordinated forming discrete complexes, whereas the Cd cations are octa­hedrally coordinated in an alternating *cis*–*cis*–*trans* and all-*trans* coordination and are linked into corrugated chains by pairs of μ-1,3-bridging seleno­cyanate anions.

## 1. Chemical context

Thio- and seleno­cyanate anions are versatile ligands because of their variable coordination modes (Buckingham, 1994 ▸; Barnett *et al.*, 2002 ▸; Werner *et al.*, 2015*a* ▸). The most common mode is the terminal coordination and μ-1,3-bridging mode, where the latter is more pronounced for chalcophilic metal cations, whereas the former dominates for less chalcophilic metal cations. For a given metal thio- or seleno­cyanate and a given mono-coordinating coligand, usually several compounds with a different ratio between the metal cation and the coligand are observed, for example *M*(NC*X*)_2_(*L*)_4_ and *M*(NC*X*)_2_(*L*)_2_, or in very few cases also *M*(NC*X*)_2_(*L*) (*M* = +2 charge transition-metal cation, *X* = S, Se and *L* = neutral mono coordinating coligand). For compounds with the composition *M*(NC*X*)_2_(*L*)_4_ and octa­hedrally coordinated metal cations mostly discrete complexes are observed and hundreds of them are reported in the literature. For ligand-deficient compounds with the composition *M*(NCS)_2_(*L*)_2_, the octa­hedral coordin­ation still dominates, but some metal ions such as Co^2+^ can show both octa­hedral and tetra­hedral coordination (Mautner *et al.*, 2018 ▸), whereas for Zn^II^, the tetra­hedral coordination is found exclusively. 
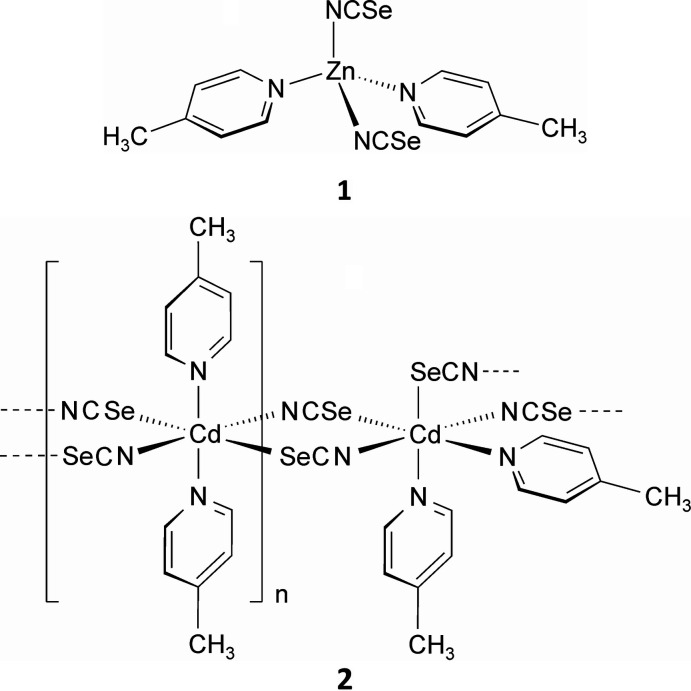


For simple geometrical considerations, compounds with the composition *M*(NC*X*)_2_(*L*)_2_ and cations that shows an octa­hedral coordination must contain μ-1,3-bridging thio or seleno­cyanate anions, and in this case the structural variability is much larger. In practically all cases they consist of *M*(NC*X*)_2_ chains or layers, but compared to chain compounds, layered structures are rare. In most of the layered compounds, the transition-metal cations are linked by single μ-1,3-bridging anionic ligands into layers (Werner *et al.*, 2015*b* ▸) or two metal cations are connected *via* pairs of anionic ligands into dinuclear units that condense into layers *via* single μ-1,3-bridging anions (Suckert *et al.*, 2016 ▸). Moreover, for an octa­hedral coordination, in principle five different isomers exist, including the all-*trans*, the all-*cis* and three *cis*–*cis*–*trans* coordinations. The majority of chain compounds show an all-*trans* coordination in which the metal cations are linked by pairs of anionic ligands, leading to the formation of linear chains (Banerjee *et al.*, 2005 ▸; Mautner *et al.*, 2018 ▸; Werner *et al.*, 2014 ▸; Rams *et al.*, 2020 ▸). Linear chains are also observed in compounds where the coligands are still in the *trans*-position, whereas the thio­cyanate N and S atoms are in the *cis*-position (Rams *et al.*, 2017 ▸; Jochim *et al.*, 2018 ▸), but there are very few examples where the coligands are in the *cis*-position, leading to the formation of corrugated chains (Banerjee *et al.*, 2005 ▸; Shi, Chen & Liu, 2006 ▸; Makhlouf *et al.*, 2022 ▸; Böhme *et al.*, 2020 ▸). Corrugated chains are also observed for an all-*cis* coordination, but only very few examples have been reported (Shi, Sun *et al.*, 2006 ▸; Zhang *et al.*, 2006 ▸; Marsh, 2009 ▸). However, all of the structure types mentioned above are well known for thio­cyanate coordination compounds, whereas the structures of seleno­cyanate compounds are not as well explored and it has not been thoroughly investigated whether compounds with thio- or seleno­cyanate anions and the same metal:coligand ratio always show the same structures and are, for example, isotypic. This might partly be traced back to the fact that some of the seleno­cyanate compounds are not very stable and that compounds with bridging anionic ligands are more difficult to prepare if less chalcophilic metal cations are used (Wriedt & Näther, 2010 ▸).

To investigate this in more detail, we prepared compounds based on Zn(NCSe)_2_ and Cd(NCSe)_2_, where the former metal ion prefers a tetra­hedral and the latter an octa­hedral coordination. Cd^II^ is also very chalcophilic, which means that compounds with bridging anionic ligands can easily be prepared. 4-Methyl­pyridine (C_6_H_7_N) was selected as coligand, for which the corresponding thio­cyanate compounds have been reported, whereas compounds with seleno­cyanate are unknown.

With Zn(NCS)_2_, compounds include three discrete complexes with the composition Zn(NCS)_2_(4-methyl­pyridine)_4_, in which the Zn cations are octa­hedrally coordinated by two terminal N-bonded thio­cyanate anions and four 4-methyl­pyridine ligands [Cambridge Structural Database (Groom *et al.*, 2016 ▸) refcodes EFESOX and YORHAO (Lipkowski *et al.*, 1994 ▸) as well as QQQBUD (Ratho & Patel, 1969 ▸)]. Two of them (EFESOX and YORHAO) represent clathrates with additional 4-methyl­pyridine mol­ecules or 4-methyl­pyridine and water mol­ecules. There is also one 4-methyl­pyridine-deficient compound with the composition Zn(NCS)_2_(4-methyl­pyridine)_2_, in which the Zn cations are tetra­hedrally coordinated by two terminal N-bonded thio­cyanate anions and two 4-methyl­pyridine ligands (refcode VONTEX; Lipkowski, 1990 ▸).

With Cd(NCS)_2_, a solvate with the composition Cd(NCS)_2_(4-methyl­pyridine)_4_·4-methyl­pyridine·water has been reported, in which the Cd cations are octa­hedrally coordinated by two terminal N-bonded seleno­cyanate anions and four 4-methyl­pyridine ligands [refcodes DEXYIO (Dyadin *et al.*, 1984 ▸), DEXYIO10, (Pervukhina *et al.*, 1986 ▸) and DEXYIO11 (Marsh, 1995 ▸)]. More importantly, two compounds with the composition Cd(NCS)_2_(4-methyl­pyridine)_2_ are found that represent isomers. In one of these, the Cd cations are octa­hedrally coordinated by two terminal N- and S-bonded seleno­cyanate anions and two 4-methyl­pyridine ligands in an all-*trans* coordination. The Cd cations are linked by pairs of seleno­cyanate anions into chains, which because of the all-*trans* coordination are linear (FAPCOO02; Neumann *et al.*, 2020 ▸). The second isomer was first reported in the triclinic space group *P* 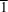 (FAPCOO; Taniguchi *et al.*, 1986 ▸) but it was later pointed out that it is better described as monoclinic, in space group *C*2/*c* (FAPCOO01; Marsh, 1995 ▸). In this compound, the Cd cations are also octa­hedrally coord­inated, linked into chains, but they are corrugated because an alternating all-*trans* and *cis*–*cis*–*trans* coordination is observed. The thermodynamic relations were determined for both isomers, indicating that they are related by monotropism with the isomer with corrugated chains as the thermodynamically stable phase (Neumann *et al.*, 2020 ▸). Finally there is one 4-methyl­pyridine-deficient compound with the composition Cd(NCS)_2_(4-methyl­pyridine), in which the Cd cations are linked by pairs of anionic ligands into chains and each two of these chains are condensed into double chains *via* μ-1,1,3-(*S*,*N*,*N*)-bridging thio­cyanate anions (refcode VUCBUT; Neumann *et al.*, 2020 ▸).

To search for new compounds related to those noted above, Zn(NO_3_)_2_·6H_2_O and Cd(NO_3_)_2_·4H_2_O were reacted with KSeCN and 4-methyl­pyridine (4-picoline)_2_, which led to the formation of two compounds with the composition Zn(NCSe)_2_(4-methyl­pyridine)_2_ (**1**) and Cd(NCeS)_2_(4-methyl­pyridine)_2_ (**2**). IR spectroscopic investigations revealed that the CN stretching vibration is located at 2072 cm^−1^ for **1** and at 2094 cm^−1^ for **2**, indicating that compound **1** contains terminally coordinated anionic ligands, whereas in **2** this value is at the borderline between that expected for a terminal and a bridging coordination (Figs. S1 and S2 in the supporting information). For both compounds, single crystals were obtained and characterized by single-crystal X-ray diffraction. Based on the crystallographic data, PXRD patterns were calculated and compared with the experimental pattern, showing that compound **1** was obtained as a pure phase, whereas compound **2** is contaminated with a very small amount of an unknown phase (Figs. S3 and S4). It is noted that even if Cd(NO_3_)_2_·4H_2_O and KSeCN are used in excess in the synthesis, there are no hints of the formation of a 4-methyl­pyridine-deficient compound with the composition Cd(NCSe)_2_(4-methyl­pyridine), as observed with Cd(NCS)_2_ (Neumann *et al.*, 2020 ▸).

## 2. Structural commentary

**Figure 1:**
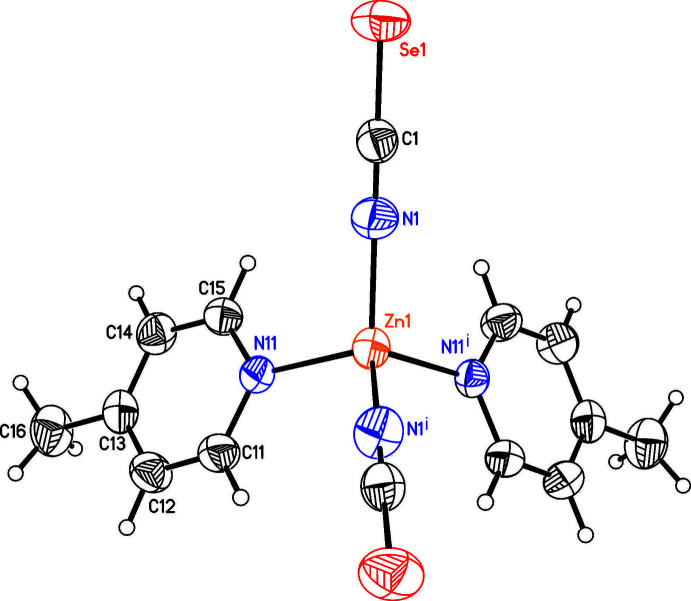
The mol­ecular structure of **1** with displacement ellipsoids drawn at the 50% probability level. Symmetry code: (i) −*x*, −*y* + 1, *z*.

The asymmetric unit of compound **1** consists of one seleno­cyanate anion and one 4-methyl­pyridine ligand in general positions, as well as one Zn^II^ cation that is located on a twofold rotation axis (Fig. 1 ▸). The Zn cations are tetra­hedrally coordinated by two symmetry-related terminal N-bonded seleno­cyanate anions and two symmetry-related 4-methyl­pyridine ligands (Fig. 1 ▸). The tetra­hedra are slightly distorted with the N_s_—Zn—N_s_ (s = seleno­cyanate) angle as the largest (Table 1 ▸). It is noted that compound **1** is isotypic to Zn(NCS)_2_(4-methyl­pyridine)_2_ reported by Lipkowski (1990 ▸).

**Figure 2:**
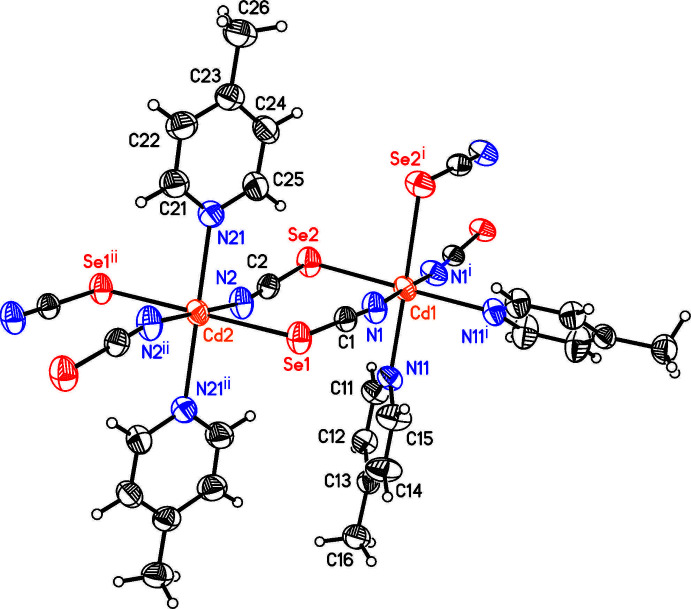
The coordination spheres of the two Cd cations in **2** with displacement ellipsoids drawn at the 50% probability level. Symmetry codes: (i) −*x* + 1, *y*, −*z* + 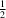 ; (ii) −*x* + 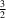 , −*y* + 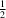 , −*z* + 1.

**Figure 3:**
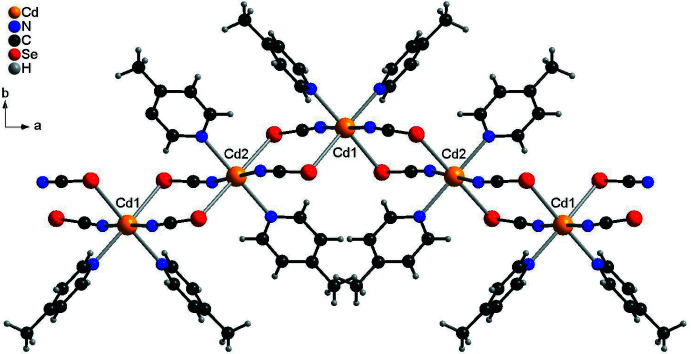
View of part of a chain in the crystal structure of compound **2** showing the alternating *cis*–*cis*–*trans* and *all*-*trans* coordination.

The asymmetric unit of compound **2** consists of two crystallographically independent Cd cations, of which Cd1 is located on a twofold rotation axis whereas Cd2 is located on a center of inversion, as well as two crystallographically independent seleno­cyanate anions and two crystallographically independent 4-methyl­pyridine ligands (Fig. 2 ▸). Both Cd cations are octa­hedrally coordinated by two N- and two S-bonding seleno­cyanate anions and two 4-methyl­pyridine ligands but Cd1 is in a *cis*–*cis*–*trans* coordination with the pyridine N atoms of the 4-methyl­pyridine ligand in the *cis* position, whereas Cd2 is in an *all*-trans coordination (Fig. 2 ▸). Both octa­hedra are slightly distorted but Cd1 is more distorted than Cd2 (Table 2 ▸). The Cd cations are linked by pairs of seleno­cyanate anions into chains that show an alternating *cis*–*cis*–*trans* and *all*-trans coordination. Because of the former, these chains are corrugated (Fig. 3 ▸).

Compound **2** is isotypic to the second isomer of Cd(NCS)_2_(4-methyl­pyridine)_2_ that crystallizes in the monoclinic space group *C*2/*c* (Marsh, 1995 ▸). In this context, it is noted that two modifications are also known for the corres­ponding Fe compound Fe(NCS)_2_(4-methyl­pyridine)_2_ (Neu­mann *et al.*, 2020 ▸), of which form **I** is isotypic to compound **2** and the corrugated chain isomer of Cd(NCS)_2_(4-methyl­pyridine)_2_, whereas form **II** of the Fe compound is isotypic to the linear chain isomer. For the Fe isomers, the same thermodynamic relations were found as for the isomers with Cd(NCS)_2_ with the corrugated chain isomer as the thermodynamically stable form (Neumann *et al.*, 2020 ▸). Moreover, compound **2** is also isotypic to Cd(NCS)_2_(4-chloro­pyridine)_2_ reported by Goher *et al.* (2003 ▸; refcode EMASIU). This can be traced back to the fact that the van der Waals radii of a methyl group and a chlorine atom are comparable, which is expressed by the so-called chloro–methyl exchange rule (Desiraju & Sarma, 1986 ▸ and references cited therein).

Finally, it is noted that some compounds with the general composition Cd(NCSe)_2_(*L*)_2_ with *L* as a monocoordinating coligand are reported, in which the Cd cations are linked by pairs of anionic ligands into chains, but the majority of compounds show an all-*trans* coordination and the formation of linear chains. An overview is given in the database survey.

## 3. Supra­molecular features

**Figure 4:**
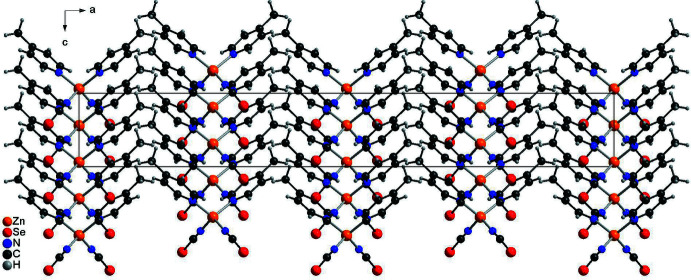
Crystal structure of compound **1** viewed along the *b*-axis direction.

In the crystal structure of compound **1**, the discrete complexes are arranged into columns that propagate along the *c*-axis direction (Fig. 4 ▸). Within these columns, the seleno­cyanate anions and the 4-methyl­pyridine ligands always point in the same direction, from which the non-centrosymmetric arrangement is visible (Fig. 4 ▸). There are no directional inter­molecular inter­actions between the complexes and nor is there any indication of π–π inter­actions.

**Figure 5:**
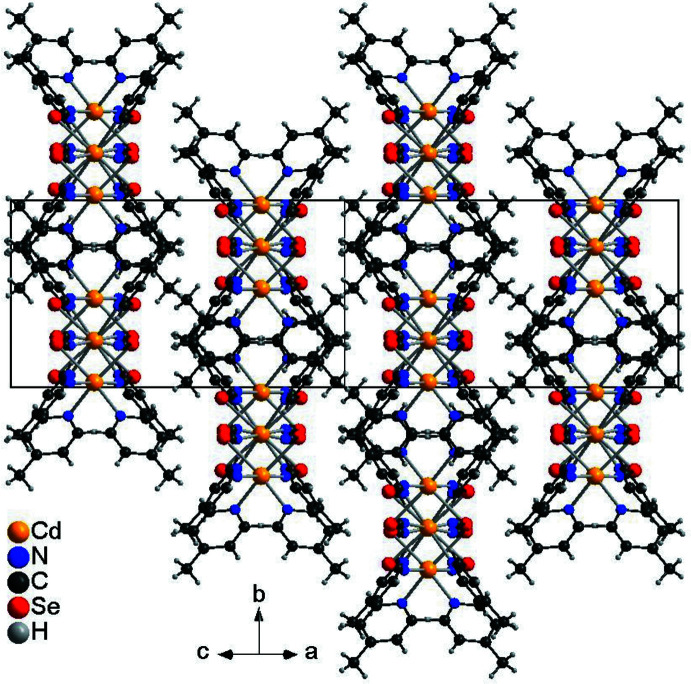
Crystal structure of compound **2** viewed along [101].

In compound **2**, the chains are closely packed and propagate along the [101] direction (Fig. 5 ▸). As in compound **1**, no pronounced inter­molecular inter­actions are observed.

## 4. Database survey

According to a search in the Cambridge Structural Database (CSD Version 5.43, March 2022; Groom *et al.*, 2016 ▸), no seleno­cyanate coordination compounds with 4-methyl­pyridine as anionic ligand have been reported but many compounds with the thio­cyanate as anion can be found. Those with Zn(NCS)_2_ and Cd(NCS)_2_ were already mentioned in the *Chemical context* section (see above).

It is also noted that several Cd(NCSe)_2_ chain compounds are reported in the CSD, but in all of them the Cd cations show an all-*trans* coordination and are linked into linear chains [BIWTOR (Fettouhi *et al.*, 2008 ▸), DAYWAE (Sadhu *et al.*, 2017 ▸), DOJBEK (Choudhury *et al.*, 2008 ▸), FAPGAG (Jess *et al.*, 2012 ▸), FIMJIW (Werner *et al.*, 2013 ▸), NAQXIO (Boeckmann, Jess *et al.*, 2011 ▸), OLOZAQ (Li & Liu, 2003 ▸), OWOHOY (Boeckmann, Reinert & Näther, 2011 ▸), QIPYAP (Secondo *et al.*, 2000 ▸) and ZANQAI (Werner *et al.*, 2012 ▸)].

However, in this context it is noted that some seleno­cyanate compounds with pyridine as coligand are found, of which those with the composition *M*(NCSe)_2_(pyridine)_2_ (*M* = Zn, Co, Ni, Cd) are of the most inter­est. The Zn compound crystallizes as discrete complexes with a tetra­hedral coordination (OWOJEQ; Boeckmann, Reinert & Näther, 2011 ▸), wheres the compounds with Fe^II^, Co^II^ and Cd^II^ crystallize as linear chains with an all-*trans* coordination [CAQVIB (Boeckmann *et al.*, 2012 ▸), ITISUA (Boeckmann & Näther, 2011 ▸)].

## 5. Synthesis and crystallization

**Synthesis**

Zn(NO_3_)_2_·6H_2_O and Cd(NO_3_)_2_·4H_2_O were purchased from Sigma Aldrich and KSeCN was purchased from Alfa Aesar. All chemicals were used without any further purification.

*Synthesis of compound **1**.*

0.5 mmol (143 mg) of Zn(NO_3_)_2_·6H_2_O and 1 mmol (144 mg) of KSeCN were reacted with 1 mmol (97.2 µl) of 4-methyl­pyridine in 2 ml of ethanol. The reaction mixture was stirred for 2 d and the colorless precipitate was filtered off, washed with a very small amount of ethanol and dried at room temperature. Single crystals were obtained from the filtrate by slow evaporation of the solvent.

*Synthesis of compound **2**.*

0.5 mmol (154 mg) of Cd(NO_3_)_2_·4H_2_O and 1 mmol (144 mg) of KSeCN were reacted with 1 mmol (97.2 µl) of 4-methyl­pyridine in 2 ml of ethanol. The reaction mixture was stirred for 2 d and the colorless precipitate was filtered off, washed with a very small amount of ethanol and dried at room temperature. Single crystals were obtained from the filtrate by slow evaporation of the solvent.

**Experimental details**

The XRPD measurements were performed with a Stoe Transmission Powder Diffraction System (STADI P) equipped with a MYTHEN 1K detector and a Johansson-type Ge(111) monochromator using Cu *K*α_1_ radiation (λ = 1.540598 Å).

The IR spectra were measured using an ATI Mattson Genesis Series FTIR Spectrometer, control software: *WINFIRST*, from ATI Mattson.

Thermogravimetry and differential thermoanalysis (TG–DTA) measurements were performed in a dynamic nitro­gen atmosphere in Al_2_O_3_ crucibles using a STA–PT 1000 thermobalance from Linseis. The instrument was calibrated using standard reference materials.

## 6. Refinement

Crystal data, data collection and structure refinement details are summarized in Table 3 ▸. C-bound H atoms were positioned with idealized geometry (C—H = 0.93–0.96 Å; methyl H atoms allowed to rotate but not to tip) and were refined isotropically with *U*_iso_(H) = 1.2*U*_eq_(C) (1.5 for methyl H atoms) using a riding model.

## Supplementary Material

Crystal structure: contains datablock(s) I, II, global. DOI: 10.1107/S2056989023000920/hb8052sup1.cif

Structure factors: contains datablock(s) I. DOI: 10.1107/S2056989023000920/hb8052Isup8.hkl

Structure factors: contains datablock(s) II. DOI: 10.1107/S2056989023000920/hb8052IIsup9.hkl

Fig. S1. IR spectrum of compound 1. Given is the value of the CN stretching vibration of the selenocyanate anions. DOI: 10.1107/S2056989023000920/hb8052sup4.jpg

Fig. S2. IR spectrum of compound 2. Given is the value of the CN stretching vibration of the selenocyanate anions. DOI: 10.1107/S2056989023000920/hb8052sup5.jpg

Fig. S3. Experimental (top) and calculated PXRD pattern (bottom) of compound 1. DOI: 10.1107/S2056989023000920/hb8052sup6.jpg

Fig. S4. Experimental (top) and calculated PXRD pattern (bottom) of compound 2. DOI: 10.1107/S2056989023000920/hb8052sup7.jpg

CCDC references: 2239352, 2239351

Additional supporting information:  crystallographic information; 3D view; checkCIF report

## Figures and Tables

**Table 1:** Selected geometric parameters (Å, °) for **1** [Chem scheme1]

Zn1—N1	1.945 (4)	Zn1—N11	2.021 (3)
			
N1—Zn1—N1^i^	120.0 (3)	N1—Zn1—N11	106.45 (15)
N1—Zn1—N11^i^	106.61 (15)	N11^i^—Zn1—N11	110.59 (18)

Symmetry code: (i) 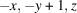 .

**Table 2:** Selected geometric parameters (Å, °) for **2** [Chem scheme1]

Cd1—N1	2.338 (3)	Cd2—N2	2.328 (4)
Cd1—N11	2.362 (4)	Cd2—N21	2.370 (4)
Cd1—Se2	2.8085 (6)	Cd2—Se1	2.8073 (5)
			
N1^i^—Cd1—N1	178.5 (2)	N2^ii^—Cd2—N2	180.0
N1—Cd1—N11^i^	92.29 (13)	N2—Cd2—N21^ii^	90.70 (14)
N1—Cd1—N11	86.63 (14)	N2—Cd2—N21	89.30 (14)
N11^i^—Cd1—N11	87.53 (18)	N21^ii^—Cd2—N21	180.0
N1^i^—Cd1—Se2	82.62 (11)	N2—Cd2—Se1^ii^	84.99 (10)
N1—Cd1—Se2	98.42 (10)	N21—Cd2—Se1^ii^	89.86 (9)
N11^i^—Cd1—Se2	169.06 (8)	N2—Cd2—Se1	95.01 (10)
N11—Cd1—Se2	90.90 (9)	N21—Cd2—Se1	90.14 (9)
Se2—Cd1—Se2^i^	92.64 (3)	Se1^ii^—Cd2—Se1	180.000 (16)

Symmetry codes: (i) 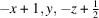 ; (ii) 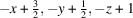 .

**Table 3:** Experimental details

	**1**	**2**
Crystal data		
Chemical formula	[Zn(NCSe)_2_(C_6_H_7_N)_2_]	[Cd(NCSe)_2_(C_6_H_7_N)_2_]
*M*_r_	461.58	508.61
Crystal system, space group	Orthorhombic, *F* *d* *d*2	Monoclinic, *C*2/*c*
Temperature (K)	293	293
*a*, *b*, *c* (Å)	37.3964 (18), 18.4780 (7), 5.1164 (2)	20.7296 (11), 9.4896 (3), 19.7364 (10)
α, β, γ (°)	90, 90, 90	90, 113.794 (3), 90
*V* (Å^3^)	3535.5 (3)	3552.5 (3)
*Z*	8	8
Radiation type	Mo *K*α	Mo *K*α
μ (mm^−1^)	5.51	5.33
Crystal size (mm)	0.25 × 0.20 × 0.20	0.18 × 0.14 × 0.10

Data collection		
Diffractometer	Stoe IPDS2	Stoe IPDS2
Absorption correction	Numerical (*X-RED* and *X-SHAPE*; Stoe, 2008 ▸)	Numerical (*X-RED* and *X-SHAPE*; Stoe, 2008 ▸)
*T*_min_, *T*_max_	0.305, 0.547	0.321, 0.446
No. of measured, independent and observed [*I* > 2σ(*I*)] reflections	14188, 1953, 1823	17056, 3469, 2911
*R*_int_	0.027	0.038
(sin θ/λ)_max_ (Å^−1^)	0.649	0.617

Refinement		
*R*[*F* ^2^ > 2σ(*F* ^2^)], *wR*(*F* ^2^), *S*	0.029, 0.067, 1.13	0.039, 0.076, 1.13
No. of reflections	1953	3469
No. of parameters	97	194
No. of restraints	1	0
H-atom treatment	H-atom parameters constrained	H-atom parameters constrained
Δρ_max_, Δρ_min_ (e Å^−3^)	0.26, −0.22	0.74, −0.63
Absolute structure	Flack *x* determined using 675 quotients [(*I* ^+^)−(*I* ^−^)]/[(*I* ^+^)+(*I* ^−^)] (Parsons et al., 2013 ▸)	–
Absolute structure parameter	0.012 (8)	–

Computer programs: *X-AREA* (Stoe, 2008 ▸), *SHELXT2014/5* (Sheldrick, 2015*a* ▸), *SHELXL2016/6* (Sheldrick, 2015*b* ▸), *DIAMOND* (Brandenburg & Putz, 1999 ▸) and *publCIF* (Westrip, 2010 ▸).

## supplementary crystallographic information

### Bis(4-methylpyridine-κN)bis(selenocyanato-κN)zinc(II) (I) Crystal data

**Table:**

[Zn(NCSe)_2_(C_6_H_7_N)_2_]	*D*_x_ = 1.734 Mg m^−^^3^
*M**_r_* = 461.58	Mo *K*α radiation, λ = 0.71073 Å
Orthorhombic, *F**d**d*2	Cell parameters from 14188 reflections
*a* = 37.3964 (18) Å	θ = 2.2–27.5°
*b* = 18.4780 (7) Å	µ = 5.51 mm^−^^1^
*c* = 5.1164 (2) Å	*T* = 293 K
*V* = 3535.5 (3) Å^3^	Block, colorless
*Z* = 8	0.25 × 0.20 × 0.20 mm
*F*(000) = 1792	

### Bis(4-methylpyridine-κN)bis(selenocyanato-κN)zinc(II) (I) Data collection

**Table:**

STOE IPDS-2 diffractometer	1823 reflections with *I* > 2σ(*I*)
ω scans	*R*_int_ = 0.027
Absorption correction: numerical (X-Red and X-Shape; Stoe, 2008)	θ_max_ = 27.5°, θ_min_ = 2.2°
*T*_min_ = 0.305, *T*_max_ = 0.547	*h* = −48→48
14188 measured reflections	*k* = −23→23
1953 independent reflections	*l* = −6→5

### Bis(4-methylpyridine-κN)bis(selenocyanato-κN)zinc(II) (I) Refinement

**Table:**

Refinement on *F*^2^	Hydrogen site location: inferred from neighbouring sites
Least-squares matrix: full	H-atom parameters constrained
*R*[*F*^2^ > 2σ(*F*^2^)] = 0.029	*w* = 1/[σ^2^(*F*_o_^2^) + (0.0306*P*)^2^ + 2.9003*P*] where *P* = (*F*_o_^2^ + 2*F*_c_^2^)/3
*wR*(*F*^2^) = 0.067	(Δ/σ)_max_ < 0.001
*S* = 1.13	Δρ_max_ = 0.26 e Å^−^^3^
1953 reflections	Δρ_min_ = −0.22 e Å^−^^3^
97 parameters	Absolute structure: Flack *x* determined using 675 quotients [(*I*^+^)-(*I*^-^)]/[(*I*^+^)+(*I*^-^)] (Parsons et al., 2013)
1 restraint	Absolute structure parameter: 0.012 (8)
Primary atom site location: dual	

### Bis(4-methylpyridine-κN)bis(selenocyanato-κN)zinc(II) (I) Special details

**Table:**

Geometry. All esds (except the esd in the dihedral angle between two l.s. planes) are estimated using the full covariance matrix. The cell esds are taken into account individually in the estimation of esds in distances, angles and torsion angles; correlations between esds in cell parameters are only used when they are defined by crystal symmetry. An approximate (isotropic) treatment of cell esds is used for estimating esds involving l.s. planes.

### Bis(4-methylpyridine-κN)bis(selenocyanato-κN)zinc(II) (I) Fractional atomic coordinates and isotropic or equivalent isotropic displacement parameters (Å^2^)

**Table:**

	*x*	*y*	*z*	*U*_iso_*/*U*_eq_	
Zn1	0.000000	0.500000	0.44116 (13)	0.05955 (17)	
Se1	0.05719 (2)	0.30585 (3)	0.91932 (12)	0.1023 (2)	
N11	0.03820 (7)	0.54594 (16)	0.2162 (7)	0.0548 (7)	
C11	0.03560 (12)	0.6142 (2)	0.1324 (10)	0.0702 (12)	
H11	0.016846	0.642476	0.193939	0.084*	
C1	0.03662 (12)	0.3754 (3)	0.7402 (9)	0.0681 (11)	
N1	0.02308 (11)	0.4217 (2)	0.6310 (9)	0.0806 (11)	
C15	0.06600 (11)	0.5073 (2)	0.1294 (9)	0.0651 (10)	
H15	0.068456	0.459745	0.185725	0.078*	
C12	0.05934 (12)	0.6443 (2)	−0.0402 (10)	0.0748 (12)	
H12	0.056342	0.691924	−0.095090	0.090*	
C16	0.11412 (15)	0.6356 (4)	−0.3232 (15)	0.0985 (16)	
H16A	0.115697	0.686987	−0.298495	0.148*	
H16B	0.106438	0.625496	−0.498454	0.148*	
H16C	0.137157	0.614117	−0.294057	0.148*	
C14	0.09088 (11)	0.5347 (2)	−0.0380 (11)	0.0734 (11)	
H14	0.110202	0.506325	−0.089267	0.088*	
C13	0.08745 (11)	0.6043 (3)	−0.1317 (9)	0.0670 (11)	

### Bis(4-methylpyridine-κN)bis(selenocyanato-κN)zinc(II) (I) Atomic displacement parameters (Å^2^)

**Table:**

	*U*^11^	*U*^22^	*U*^33^	*U*^12^	*U*^13^	*U*^23^
Zn1	0.0556 (3)	0.0672 (3)	0.0558 (3)	−0.0044 (3)	0.000	0.000
Se1	0.1184 (4)	0.0790 (3)	0.1095 (5)	0.0235 (3)	−0.0162 (4)	0.0132 (4)
N11	0.0497 (14)	0.0573 (16)	0.0573 (19)	−0.0027 (12)	−0.0047 (14)	0.0011 (15)
C11	0.065 (2)	0.064 (2)	0.081 (3)	0.0077 (18)	0.007 (2)	0.006 (2)
C1	0.069 (2)	0.073 (3)	0.063 (3)	−0.007 (2)	−0.002 (2)	0.002 (2)
N1	0.081 (2)	0.088 (3)	0.073 (3)	0.002 (2)	−0.007 (2)	0.017 (2)
C15	0.063 (2)	0.0566 (19)	0.076 (3)	0.0029 (16)	0.0013 (19)	−0.001 (2)
C12	0.080 (3)	0.069 (2)	0.075 (3)	−0.001 (2)	0.008 (3)	0.013 (2)
C16	0.090 (3)	0.120 (4)	0.085 (3)	−0.019 (3)	0.021 (3)	0.015 (4)
C14	0.063 (2)	0.076 (3)	0.081 (3)	0.0048 (18)	0.011 (2)	−0.007 (3)
C13	0.064 (2)	0.079 (3)	0.057 (3)	−0.014 (2)	0.0001 (18)	−0.0001 (19)

### Bis(4-methylpyridine-κN)bis(selenocyanato-κN)zinc(II) (I) Geometric parameters (Å, º)

**Table:**

Zn1—N1	1.945 (4)	C15—C14	1.362 (7)
Zn1—N1^i^	1.945 (4)	C15—H15	0.9300
Zn1—N11^i^	2.021 (3)	C12—C13	1.368 (6)
Zn1—N11	2.021 (3)	C12—H12	0.9300
Se1—C1	1.756 (5)	C16—C13	1.513 (7)
N11—C11	1.335 (5)	C16—H16A	0.9600
N11—C15	1.337 (5)	C16—H16B	0.9600
C11—C12	1.371 (6)	C16—H16C	0.9600
C11—H11	0.9300	C14—C13	1.379 (6)
C1—N1	1.140 (5)	C14—H14	0.9300
			
N1—Zn1—N1^i^	120.0 (3)	C14—C15—H15	118.6
N1—Zn1—N11^i^	106.61 (15)	C13—C12—C11	119.9 (4)
N1^i^—Zn1—N11^i^	106.44 (15)	C13—C12—H12	120.1
N1—Zn1—N11	106.45 (15)	C11—C12—H12	120.1
N1^i^—Zn1—N11	106.61 (15)	C13—C16—H16A	109.5
N11^i^—Zn1—N11	110.59 (18)	C13—C16—H16B	109.5
C11—N11—C15	117.0 (4)	H16A—C16—H16B	109.5
C11—N11—Zn1	121.9 (3)	C13—C16—H16C	109.5
C15—N11—Zn1	121.0 (3)	H16A—C16—H16C	109.5
N11—C11—C12	122.9 (4)	H16B—C16—H16C	109.5
N11—C11—H11	118.5	C15—C14—C13	120.1 (4)
C12—C11—H11	118.5	C15—C14—H14	120.0
N1—C1—Se1	177.9 (4)	C13—C14—H14	120.0
C1—N1—Zn1	179.3 (4)	C12—C13—C14	117.2 (4)
N11—C15—C14	122.9 (4)	C12—C13—C16	121.4 (4)
N11—C15—H15	118.6	C14—C13—C16	121.3 (5)

Symmetry code: (i) −*x*, −*y*+1, *z*.

### catena-Poly[[bis(4-methylpyridine-κN)cadmium(II)]-di-µ-selenocyanato-κ2N:Se;κ2Se:N] (II) Crystal data

**Table:**

[Cd(NCSe)_2_(C_6_H_7_N)_2_]	*F*(000) = 1936
*M**_r_* = 508.61	*D*_x_ = 1.902 Mg m^−^^3^
Monoclinic, *C*2/*c*	Mo *K*α radiation, λ = 0.71073 Å
*a* = 20.7296 (11) Å	Cell parameters from 17056 reflections
*b* = 9.4896 (3) Å	θ = 2.2–26.0°
*c* = 19.7364 (10) Å	µ = 5.33 mm^−^^1^
β = 113.794 (3)°	*T* = 293 K
*V* = 3552.5 (3) Å^3^	Block, colorless
*Z* = 8	0.18 × 0.14 × 0.10 mm

### catena-Poly[[bis(4-methylpyridine-κN)cadmium(II)]-di-µ-selenocyanato-κ2N:Se;κ2Se:N] (II) Data collection

**Table:**

STOE IPDS-2 diffractometer	2911 reflections with *I* > 2σ(*I*)
ω scans	*R*_int_ = 0.038
Absorption correction: numerical (X-Red and X-Shape; Stoe, 2008)	θ_max_ = 26.0°, θ_min_ = 2.2°
*T*_min_ = 0.321, *T*_max_ = 0.446	*h* = −25→25
17056 measured reflections	*k* = −8→11
3469 independent reflections	*l* = −24→24

### catena-Poly[[bis(4-methylpyridine-κN)cadmium(II)]-di-µ-selenocyanato-κ2N:Se;κ2Se:N] (II) Refinement

**Table:**

Refinement on *F*^2^	Primary atom site location: dual
Least-squares matrix: full	Hydrogen site location: inferred from neighbouring sites
*R*[*F*^2^ > 2σ(*F*^2^)] = 0.039	H-atom parameters constrained
*wR*(*F*^2^) = 0.076	*w* = 1/[σ^2^(*F*_o_^2^) + (0.0229*P*)^2^ + 7.6388*P*] where *P* = (*F*_o_^2^ + 2*F*_c_^2^)/3
*S* = 1.13	(Δ/σ)_max_ = 0.001
3469 reflections	Δρ_max_ = 0.74 e Å^−^^3^
194 parameters	Δρ_min_ = −0.63 e Å^−^^3^
0 restraints	

### catena-Poly[[bis(4-methylpyridine-κN)cadmium(II)]-di-µ-selenocyanato-κ2N:Se;κ2Se:N] (II) Special details

**Table:**

Geometry. All esds (except the esd in the dihedral angle between two l.s. planes) are estimated using the full covariance matrix. The cell esds are taken into account individually in the estimation of esds in distances, angles and torsion angles; correlations between esds in cell parameters are only used when they are defined by crystal symmetry. An approximate (isotropic) treatment of cell esds is used for estimating esds involving l.s. planes.

### catena-Poly[[bis(4-methylpyridine-κN)cadmium(II)]-di-µ-selenocyanato-κ2N:Se;κ2Se:N] (II) Fractional atomic coordinates and isotropic or equivalent isotropic displacement parameters (Å^2^)

**Table:**

	*x*	*y*	*z*	*U*_iso_*/*U*_eq_	
Cd1	0.500000	0.47303 (5)	0.250000	0.05827 (14)	
Cd2	0.750000	0.250000	0.500000	0.05746 (13)	
N1	0.5569 (2)	0.4762 (5)	0.37921 (19)	0.0724 (11)	
C1	0.5986 (2)	0.4628 (4)	0.4381 (2)	0.0539 (10)	
Se1	0.66318 (2)	0.44348 (6)	0.53116 (2)	0.06746 (15)	
N2	0.6944 (2)	0.2763 (5)	0.3720 (2)	0.0715 (11)	
C2	0.6512 (2)	0.2729 (5)	0.3135 (2)	0.0582 (10)	
Se2	0.58388 (3)	0.26863 (6)	0.22157 (3)	0.07812 (18)	
N11	0.57906 (18)	0.6528 (4)	0.2507 (2)	0.0640 (9)	
C11	0.6021 (2)	0.6648 (5)	0.1971 (2)	0.0700 (12)	
H11	0.585089	0.601802	0.157671	0.084*	
C12	0.6496 (3)	0.7651 (5)	0.1968 (3)	0.0679 (12)	
H12	0.663234	0.769834	0.157369	0.081*	
C13	0.6773 (2)	0.8592 (5)	0.2548 (3)	0.0629 (11)	
C14	0.6536 (3)	0.8466 (6)	0.3102 (3)	0.0938 (18)	
H14	0.670037	0.907735	0.350437	0.113*	
C15	0.6059 (3)	0.7438 (6)	0.3062 (3)	0.0909 (18)	
H15	0.591248	0.737234	0.344883	0.109*	
C16	0.7307 (3)	0.9687 (6)	0.2579 (3)	0.0824 (15)	
H16A	0.776309	0.925625	0.273302	0.124*	
H16B	0.732202	1.040818	0.292621	0.124*	
H16C	0.717741	1.009812	0.209722	0.124*	
N21	0.67039 (19)	0.0657 (4)	0.4930 (2)	0.0641 (9)	
C21	0.6897 (3)	−0.0359 (6)	0.5440 (3)	0.0803 (15)	
H21	0.736013	−0.036657	0.578951	0.096*	
C22	0.6451 (3)	−0.1392 (6)	0.5477 (3)	0.0844 (15)	
H22	0.661708	−0.207526	0.584525	0.101*	
C23	0.5759 (3)	−0.1424 (5)	0.4974 (3)	0.0708 (13)	
C24	0.5563 (3)	−0.0392 (6)	0.4441 (3)	0.0744 (13)	
H24	0.510543	−0.037549	0.407877	0.089*	
C25	0.6039 (3)	0.0619 (6)	0.4438 (3)	0.0736 (13)	
H25	0.588663	0.130929	0.407229	0.088*	
C26	0.5257 (3)	−0.2518 (6)	0.5017 (4)	0.0890 (17)	
H26A	0.479146	−0.212412	0.484738	0.133*	
H26B	0.540300	−0.282538	0.552103	0.133*	
H26C	0.525360	−0.330752	0.471169	0.133*	

### catena-Poly[[bis(4-methylpyridine-κN)cadmium(II)]-di-µ-selenocyanato-κ2N:Se;κ2Se:N] (II) Atomic displacement parameters (Å^2^)

**Table:**

	*U*^11^	*U*^22^	*U*^33^	*U*^12^	*U*^13^	*U*^23^
Cd1	0.0473 (2)	0.0720 (3)	0.0452 (2)	0.000	0.00794 (18)	0.000
Cd2	0.0497 (2)	0.0680 (3)	0.0476 (2)	0.0026 (2)	0.01232 (19)	0.00515 (19)
N1	0.061 (2)	0.095 (3)	0.045 (2)	0.012 (2)	0.0049 (17)	−0.0056 (19)
C1	0.051 (2)	0.058 (3)	0.052 (2)	0.0048 (18)	0.021 (2)	−0.0023 (18)
Se1	0.0620 (3)	0.0856 (4)	0.0431 (2)	0.0160 (2)	0.0091 (2)	−0.0016 (2)
N2	0.068 (2)	0.097 (3)	0.044 (2)	0.018 (2)	0.0162 (18)	0.0062 (19)
C2	0.062 (3)	0.059 (3)	0.055 (2)	0.008 (2)	0.025 (2)	0.0001 (19)
Se2	0.0686 (3)	0.0937 (4)	0.0547 (3)	0.0124 (3)	0.0068 (2)	−0.0188 (2)
N11	0.056 (2)	0.078 (3)	0.059 (2)	−0.0090 (19)	0.0253 (17)	−0.0172 (19)
C11	0.070 (3)	0.083 (3)	0.056 (2)	−0.013 (3)	0.024 (2)	−0.019 (2)
C12	0.071 (3)	0.077 (3)	0.061 (3)	−0.003 (2)	0.033 (2)	−0.010 (2)
C13	0.054 (2)	0.065 (3)	0.070 (3)	−0.001 (2)	0.025 (2)	−0.012 (2)
C14	0.113 (4)	0.100 (4)	0.084 (4)	−0.043 (4)	0.057 (3)	−0.045 (3)
C15	0.110 (4)	0.102 (4)	0.084 (4)	−0.035 (4)	0.063 (3)	−0.039 (3)
C16	0.077 (3)	0.081 (4)	0.096 (4)	−0.012 (3)	0.042 (3)	−0.018 (3)
N21	0.061 (2)	0.069 (2)	0.062 (2)	0.0001 (19)	0.0245 (18)	0.0037 (19)
C21	0.073 (3)	0.079 (4)	0.073 (3)	−0.007 (3)	0.014 (3)	0.007 (3)
C22	0.088 (4)	0.074 (3)	0.082 (3)	−0.009 (3)	0.025 (3)	0.006 (3)
C23	0.076 (3)	0.064 (3)	0.082 (3)	−0.006 (3)	0.042 (3)	−0.019 (3)
C24	0.054 (3)	0.082 (4)	0.083 (3)	−0.005 (2)	0.023 (2)	−0.014 (3)
C25	0.060 (3)	0.082 (3)	0.074 (3)	0.004 (3)	0.022 (2)	0.003 (3)
C26	0.089 (4)	0.080 (4)	0.114 (5)	−0.018 (3)	0.058 (4)	−0.022 (3)

### catena-Poly[[bis(4-methylpyridine-κN)cadmium(II)]-di-µ-selenocyanato-κ2N:Se;κ2Se:N] (II) Geometric parameters (Å, º)

**Table:**

Cd1—N1^i^	2.338 (3)	C13—C14	1.373 (6)
Cd1—N1	2.338 (3)	C13—C16	1.501 (7)
Cd1—N11^i^	2.362 (4)	C14—C15	1.370 (7)
Cd1—N11	2.362 (4)	C14—H14	0.9300
Cd1—Se2	2.8085 (6)	C15—H15	0.9300
Cd1—Se2^i^	2.8086 (6)	C16—H16A	0.9600
Cd2—N2^ii^	2.328 (4)	C16—H16B	0.9600
Cd2—N2	2.328 (4)	C16—H16C	0.9600
Cd2—N21^ii^	2.370 (4)	N21—C25	1.329 (6)
Cd2—N21	2.370 (4)	N21—C21	1.332 (6)
Cd2—Se1^ii^	2.8073 (5)	C21—C22	1.369 (7)
Cd2—Se1	2.8073 (5)	C21—H21	0.9300
N1—C1	1.142 (5)	C22—C23	1.378 (7)
C1—Se1	1.793 (4)	C22—H22	0.9300
N2—C2	1.141 (5)	C23—C24	1.373 (7)
C2—Se2	1.788 (5)	C23—C26	1.497 (7)
N11—C15	1.329 (6)	C24—C25	1.377 (7)
N11—C11	1.329 (5)	C24—H24	0.9300
C11—C12	1.372 (6)	C25—H25	0.9300
C11—H11	0.9300	C26—H26A	0.9600
C12—C13	1.381 (6)	C26—H26B	0.9600
C12—H12	0.9300	C26—H26C	0.9600
			
N1^i^—Cd1—N1	178.5 (2)	C11—C12—C13	120.3 (4)
N1^i^—Cd1—N11^i^	86.63 (14)	C11—C12—H12	119.8
N1—Cd1—N11^i^	92.29 (13)	C13—C12—H12	119.8
N1^i^—Cd1—N11	92.30 (13)	C14—C13—C12	116.2 (4)
N1—Cd1—N11	86.63 (14)	C14—C13—C16	121.5 (4)
N11^i^—Cd1—N11	87.53 (18)	C12—C13—C16	122.3 (4)
N1^i^—Cd1—Se2	82.62 (11)	C15—C14—C13	119.9 (5)
N1—Cd1—Se2	98.42 (10)	C15—C14—H14	120.0
N11^i^—Cd1—Se2	169.06 (8)	C13—C14—H14	120.0
N11—Cd1—Se2	90.90 (9)	N11—C15—C14	124.2 (5)
N1^i^—Cd1—Se2^i^	98.42 (10)	N11—C15—H15	117.9
N1—Cd1—Se2^i^	82.62 (11)	C14—C15—H15	117.9
N11^i^—Cd1—Se2^i^	90.90 (9)	C13—C16—H16A	109.5
N11—Cd1—Se2^i^	169.06 (8)	C13—C16—H16B	109.5
Se2—Cd1—Se2^i^	92.64 (3)	H16A—C16—H16B	109.5
N2^ii^—Cd2—N2	180.0	C13—C16—H16C	109.5
N2^ii^—Cd2—N21^ii^	89.30 (14)	H16A—C16—H16C	109.5
N2—Cd2—N21^ii^	90.70 (14)	H16B—C16—H16C	109.5
N2^ii^—Cd2—N21	90.70 (14)	C25—N21—C21	116.0 (4)
N2—Cd2—N21	89.30 (14)	C25—N21—Cd2	123.7 (3)
N21^ii^—Cd2—N21	180.0	C21—N21—Cd2	120.0 (3)
N2^ii^—Cd2—Se1^ii^	95.00 (10)	N21—C21—C22	123.7 (5)
N2—Cd2—Se1^ii^	84.99 (10)	N21—C21—H21	118.1
N21^ii^—Cd2—Se1^ii^	90.14 (9)	C22—C21—H21	118.1
N21—Cd2—Se1^ii^	89.86 (9)	C21—C22—C23	120.4 (5)
N2^ii^—Cd2—Se1	85.00 (10)	C21—C22—H22	119.8
N2—Cd2—Se1	95.01 (10)	C23—C22—H22	119.8
N21^ii^—Cd2—Se1	89.86 (9)	C24—C23—C22	115.8 (5)
N21—Cd2—Se1	90.14 (9)	C24—C23—C26	122.8 (5)
Se1^ii^—Cd2—Se1	180.000 (16)	C22—C23—C26	121.4 (5)
C1—N1—Cd1	161.8 (4)	C23—C24—C25	120.7 (5)
N1—C1—Se1	179.0 (4)	C23—C24—H24	119.7
C1—Se1—Cd2	97.03 (13)	C25—C24—H24	119.7
C2—N2—Cd2	159.7 (4)	N21—C25—C24	123.3 (5)
N2—C2—Se2	179.5 (5)	N21—C25—H25	118.4
C2—Se2—Cd1	94.25 (14)	C24—C25—H25	118.4
C15—N11—C11	115.8 (4)	C23—C26—H26A	109.5
C15—N11—Cd1	122.2 (3)	C23—C26—H26B	109.5
C11—N11—Cd1	121.9 (3)	H26A—C26—H26B	109.5
N11—C11—C12	123.5 (4)	C23—C26—H26C	109.5
N11—C11—H11	118.3	H26A—C26—H26C	109.5
C12—C11—H11	118.3	H26B—C26—H26C	109.5

Symmetry codes: (i) −*x*+1, *y*, −*z*+1/2; (ii) −*x*+3/2, −*y*+1/2, −*z*+1.
