# Supplementary figures and images for: Syntheses and crystal structures of bis­(4-methyl­pyridine-κN)bis­(seleno­cyanato-κN)zinc(II) and catena-poly[[bis­(4-methyl­pyridine-κN)cadmium(II)]-di-μ-seleno­cyanato-κ2 N:Se;κ2 Se:N]
Source: Acta Crystallogr E Crystallogr Commun. 2023 Feb 7;79(Pt 3):136–41. doi: 10.1107/S2056989023000920 (PMC9993905; doi:10.1107/S2056989023000920)

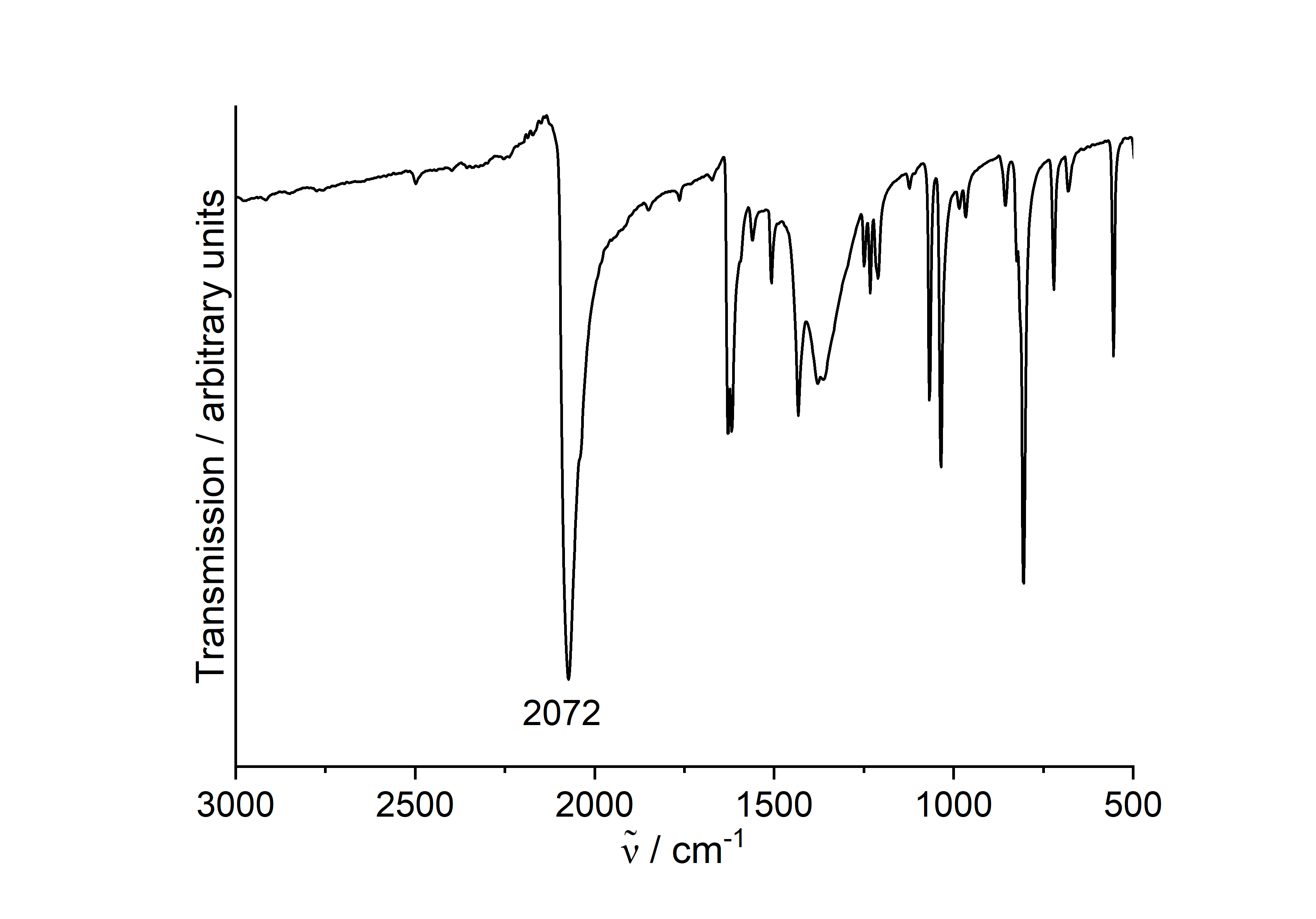

Supplement: Supplementary file 4 [file e-79-00136-sup4.jpg]

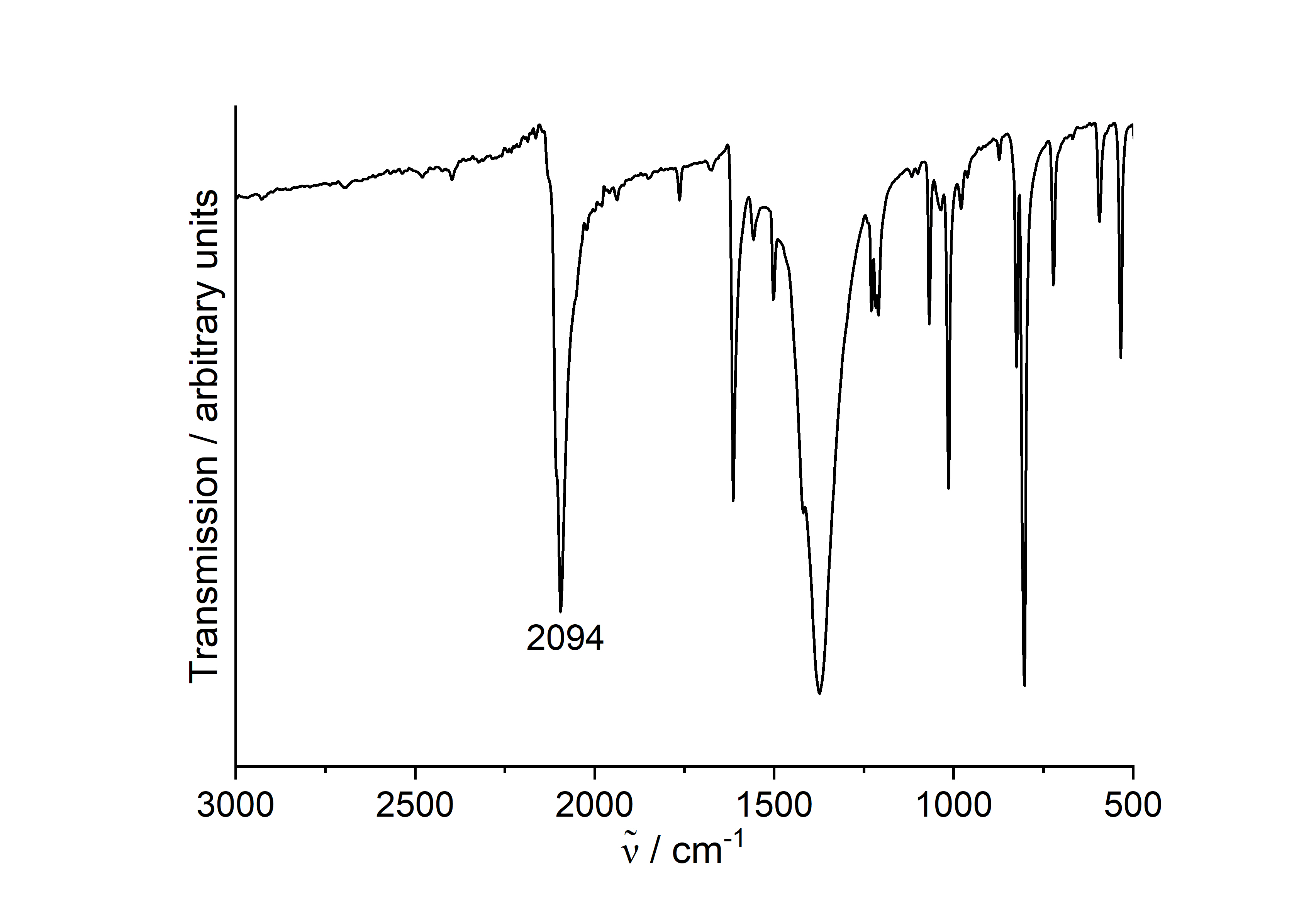

Supplement: Supplementary file 5 [file e-79-00136-sup5.jpg]

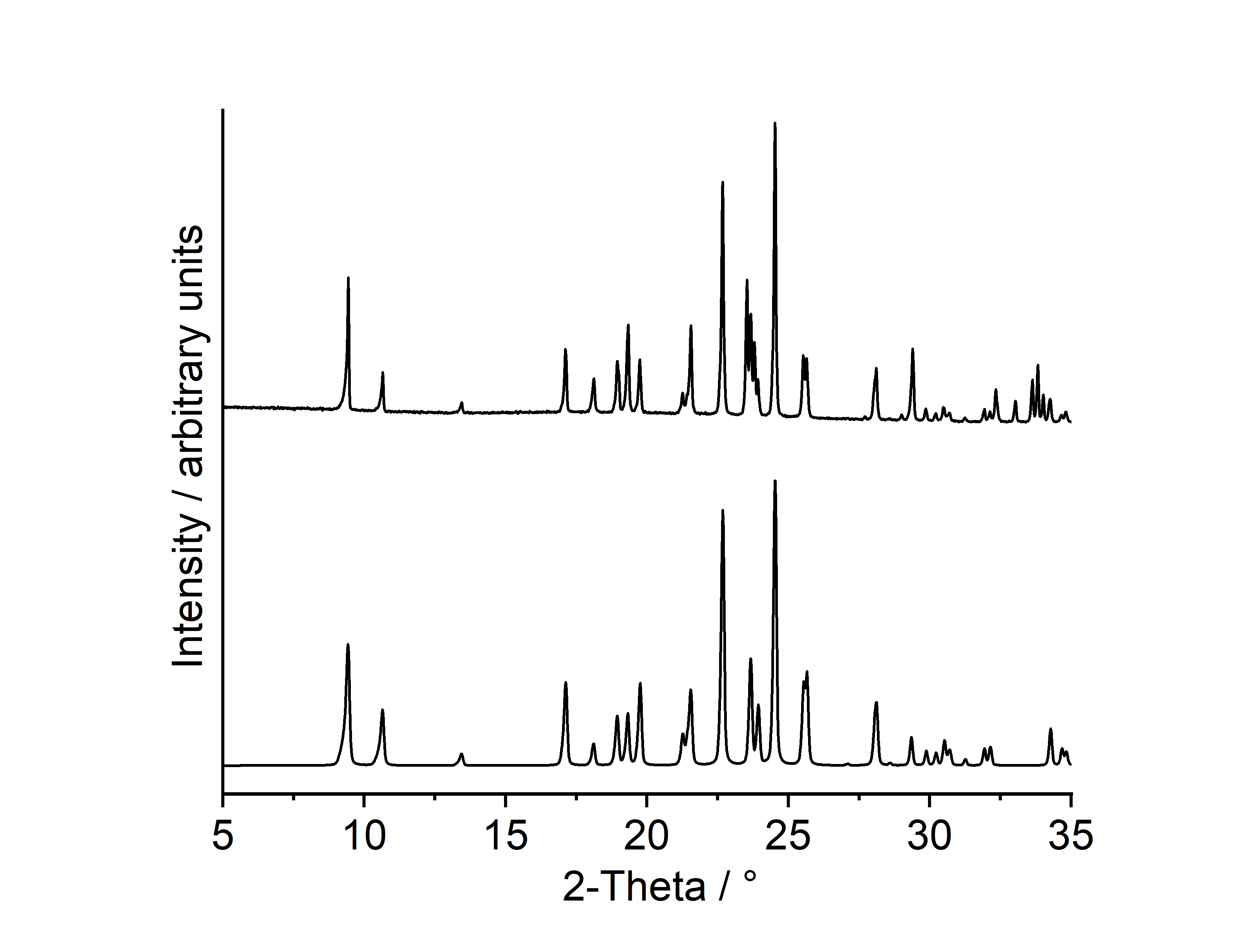

Supplement: Supplementary file 6 [file e-79-00136-sup6.jpg]

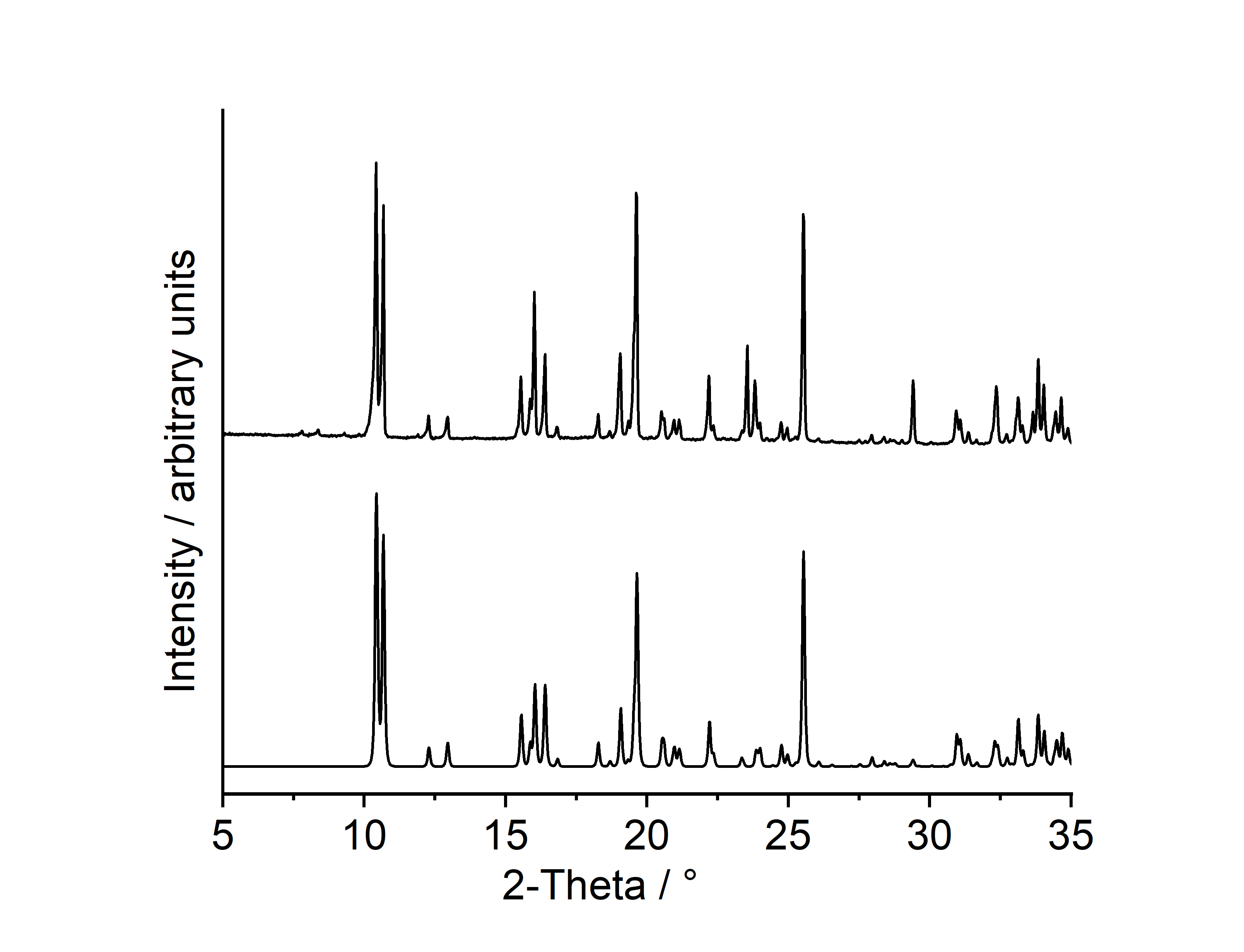

Supplement: Supplementary file 7 [file e-79-00136-sup7.jpg]
